# Navigating Through the Diagnostic Challenges Involved in Artery of Percheron Infarction in a Young Stroke Patient

**DOI:** 10.7759/cureus.78378

**Published:** 2025-02-02

**Authors:** Jaziya Jabeen, Siju J Koonan, Fiju Chacko, Jazeem A Ahamed Kabir

**Affiliations:** 1 Cardiology, Royal Cornwall Hospital, Cornwall, GBR; 2 Internal Medicine, Jubilee Mission Medical College and Research Institute, Thrissur, IND; 3 Neurology, Jubilee Mission Medical College and Research Institute, Thrissur, IND; 4 General Practice, Royal Cornwall Hospital, Cornwall, GBR

**Keywords:** aop infarct, artery of percheron, diagnostics, posterior circulation stroke, rare anatomical variant

## Abstract

Artery of Percheron (AOP) infarction is a rare but clinically important cause of unilateral or bilateral thalamic infarction, commonly with a heterogeneous presentation and nonspecific neurological symptoms. In contrast to unilateral thalamic strokes, AOP infarction can present a unique hurdle for diagnostic considerations owing to its atypical clinical manifestation and the lack of widespread knowledge of this anatomic variant among clinicians. AOP infarction is associated with potentially better outcomes if it is diagnosed early, as timely and appropriate interventions can greatly affect the outcome.

This case of a 39-year-old Indian female emphasizes the necessity of taking AOP infarction into account in the differential diagnosis of patients with acute symptoms of central nervous system dysfunction, particularly when initial imaging is nondiagnostic or when clinical features such as altered mental status, visual disturbances, or cognitive impairment are present. This highlights the challenges encountered in diagnosing and treating the condition while also necessitating a thorough history-taking and study of symptomatology. Early diagnosis, the use of appropriate imaging techniques, and timely treatment help increase the chances of good functional outcomes and reduce long-term neurological deficits, highlighting the need for multidisciplinary care.

## Introduction

The artery of Percheron (AOP) is a variant of the normal anatomy of the thalamic and midbrain circulation and was first described by Gérard Percheron in 1973 [1]. It is one of the four anatomical variants described for the thalamus and the midbrain. The most common type is type I. Both the paramedian arteries arise from the proximal or P1 segment of each posterior cerebral artery in type I. Type 2 has two further divisions: in type IIa, two paramedian arteries emerge from either the right or the left posterior cerebral artery, whereas in type IIb, which is also known as the artery of Percheron, a single trunk arising from the P1 segment later bifurcates to supply both the paramedian thalamus and the rostral midbrain. Type III involves a communicating artery between the paramedian arteries from the P1 segments of the bilateral posterior cerebral arteries [1,2,3].

The AOP, in approximately one-third of humans, arises as a single arterial trunk that then bifurcates into separate arteries to perfuse the bilateral paramedian thalami. This configuration is found in 4% to 18% of the population, and its actual percentage is yet to be determined since it cannot be easily recognized via conventional imaging [3,4].

The distinctive vascular architecture of the AOP renders it especially susceptible to ischemic injury due to embolism, thrombosis, or systemic hypoperfusion [5]. Clinically, the presentation varies from mild cognitive impairment, drowsiness, and sensory and visual defects to life-threatening situations such as coma, akinetic mutism, and locked-in syndrome [5].

Thalamic and midbrain involvement also leads to one of the classical presentations of AOP infarction: the triad of altered mental status, vertical gaze palsy, and memory disturbances. However, this triad is not always present, and atypical or incomplete symptoms can lead to a delay in diagnosis [5-7]. Bilateral thalamic infarction can also mimic other pathological conditions, specifically top-of-the-basilar syndrome and Wernicke's encephalopathy [7].

The outcome of AOP infarction is highly variable depending on the degree of neurological insult and the timing of intervention. Although many patients recover with only minor residual deficits, others are left with debilitating long-term impairments in cognition, motor function, and quality of life [7]. Early diagnosis is essential for better outcomes and for reducing the risk of complications for this rare condition.

We present a case of AOP infarct in an adult female with a significant family history of cardiovascular disease. This case illustrates the diagnostic challenge of AOP infarction and emphasizes awareness of this rare entity when considering the differential diagnosis of bilateral thalamic syndromes, even in young stroke patients.

## Case presentation

A 39-year-old Indian female, a known case of bronchial asthma, presented to the ED with a history of blurring of vision followed by generalized tiredness and a decreased response that lasted for approximately five minutes. Family history revealed a history of stroke in her father and a history of myocardial infarction in her mother. On examination, the patient was drowsy, with a Glasgow Coma Scale (GCS) of 13/15 E4V4M5. The pupils were 3 mm bilaterally and sluggishly reactive. Vitals were as follows: pulse rate (PR) 69/min, blood pressure (BP) 114/72 mmHg, oxygen saturation (SpO2) 99% in room air, a temperature of 98.6°F, and general random blood sugar (GRBS) of 134. The electrocardiogram (ECG) had no significant abnormalities. A plain CT brain scan was taken and did not reveal any obvious abnormality in the brain parenchyma. The basic metabolic panel and blood tests were within normal limits (Table 1). The fasting blood sugar and lipid profiles from a previous hospital were also within normal limits. 

**Table 1:** Routine investigation parameter results CRP: C-reactive protein; SGOT: serum glutamic oxaloacetic transaminase; SGPT: serum glutamic pyruvic transaminase; PT/INR: prothrombin time/international normalized ratio; APTT: activated partial thromboplastin time

Parameters	Reference range	Day 1
Hemoglobin	12-14 g/dL	12.7
RBC count	4-5 x 10^6^/µl	4.30
Total WBC count	4-11x10^3^/µl	6.97
Differential count		N53 L34
Platelet count	150-400x10^3^/µl	170
CRP		0.13
SGOT	14-36U/L	23
SGPT	<35U/L	8
Blood urea	15-36 mg/dL	11
S. creatinine	0.52-1.04 mg/dL	0.7
PT/INR	Control-12.1/INR 0.96-1.23	11.6/0.96
APTT	Control 30.0	32.6
D-dimer	<250 mg/dL	358
S. Calcium	8.8-10.6 mg/dL	8.3
S. Magnesium	1.62-3 mg/dL	2.3
Sodium	137-145 mEq/L	138
Potassium	3.5-5.1 mEq/L	3.8
Protein	6.3-8.2 g/dL	7.7
Troponin I		<0.003
Lupus anticoagulant	Negative <1.2	0.86

Neurology consultation was sought. Intravenous brivaracetam 100 mg immediately (STAT) was given considering a probable diagnosis of seizure, which was suggestive from the history. The patient was admitted to the medical ward for further evaluation and management. MRI and EEG were also suggested.

**Figure 1:**
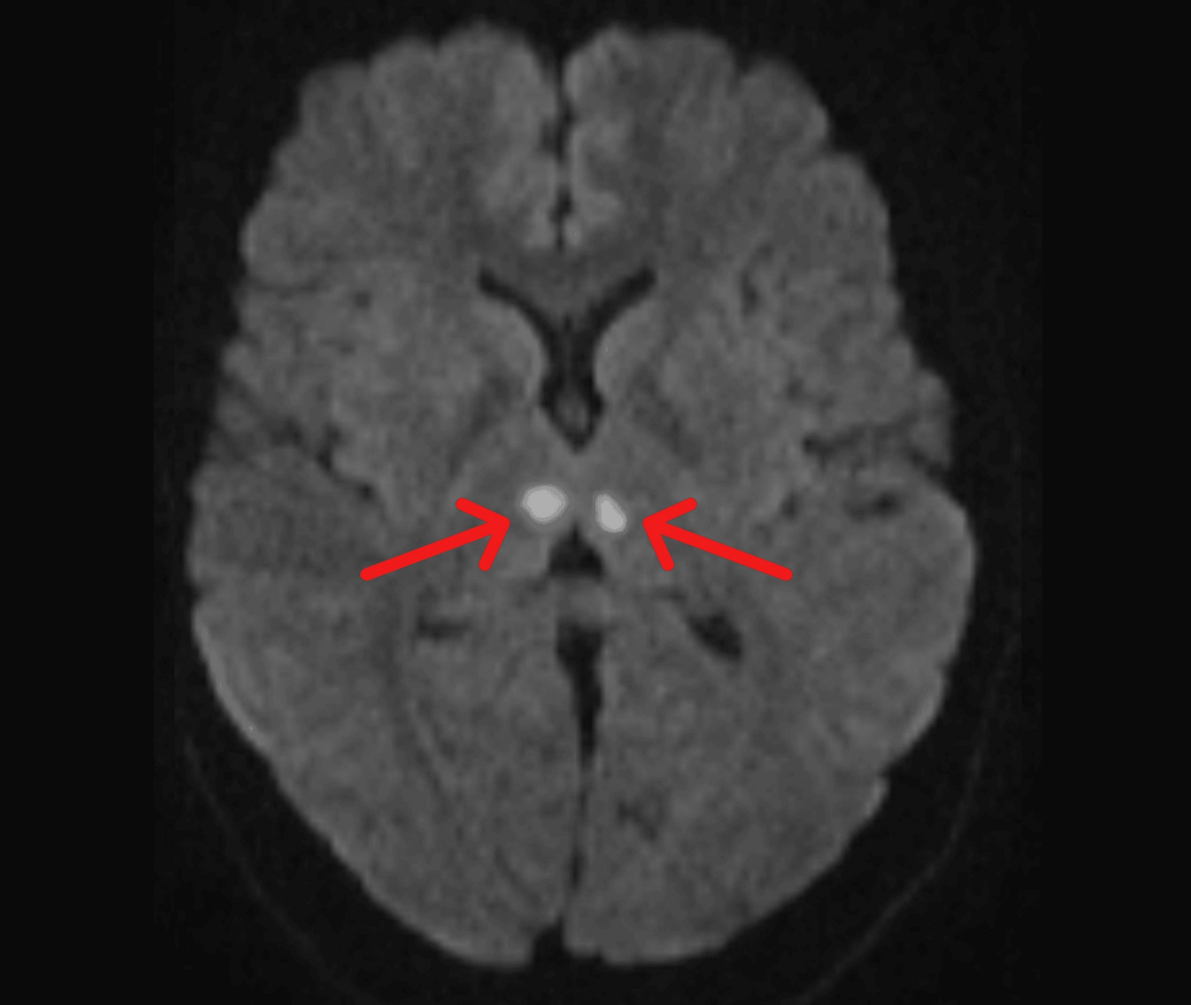
Axial FLAIR at the level of basal ganglia showing FLAIR hyperintense focus in bilateral medial thalami FLAIR: fluid-attenuated inversion recovery

**Figure 2:**
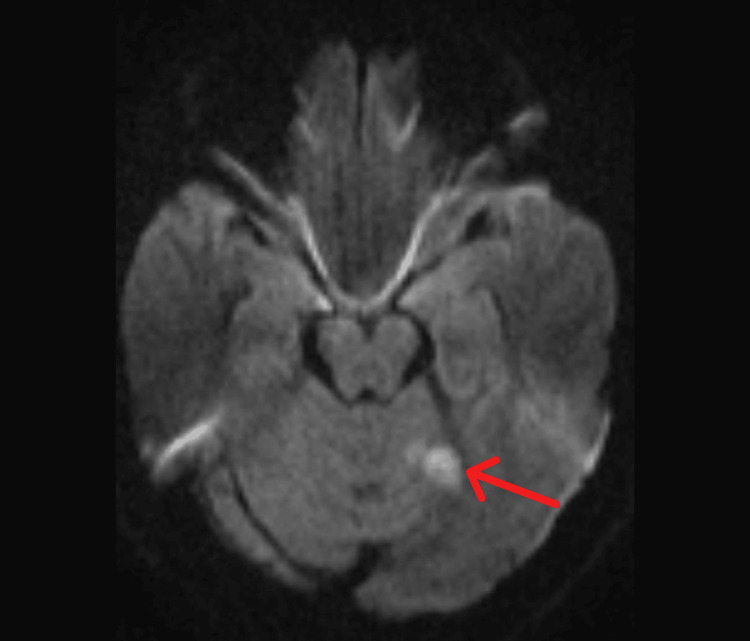
Axial FLAIR at the level of the inferior collicular region showing a patchy area of FLAIR hyperintensity in the left superior cerebellum FLAIR: fluid-attenuated inversion recovery

On day two, the patient showed significant improvement and was conscious and oriented. On further examination, extraocular movements were full, and the pupils were equal and reactive. The GCS was 15/15. Diffusion-weighted magnetic resonance imaging (DW-MRI) of the brain done on the next day revealed symmetrical subcentimetric foci of diffusion restriction with corresponding fluid-attenuated inversion recovery (FLAIR) hyperintensity in the bilateral medial thalami (Figure 1). A separate focus of diffusion restriction in the left superior cerebellum (Figure 2) was seen. All of these features favored an acute infarct, involving the artery of Percheron. No hemorrhage was detected. 

Given the MRI findings, the patient was started on T. ecospirin 75 mg and T. atorvastatin 20 mg once daily [8]. Since the therapeutic window for thrombolysis had already been exceeded, a conservative management plan was decided upon and consisted of monitoring neurological status, hydration, and supportive care.

On day three, a complete neurological examination was performed. Bulk and tone were normal. A power of 5/5 was elicited for both the upper and lower limbs. No neck stiffness was present. Sensory system examination revealed a loss of sensation to pain and temperature in both lower limbs. The rest of the neurological examination was unremarkable. The other systems examined were within normal limits.

Further, the electroencephalogram (EEG) done was found to be normal, whereas the echocardiogram revealed moderate tricuspid regurgitation and mild pulmonary artery hypertension. 

On day four, time-of-flight (TOF) magnetic resonance (MR) angiography was done and revealed all major cervical and intracranial arteries patent with normal flow-related enhancement. No significant aneurysms or stenosis were detected.

While a full workup, including fasting blood sugar (FBS), lipid profile, thyroid function tests, and investigations for hypercoagulable states as seen in young stroke patients [9], was planned in the coming days, the patient requested discharge on day four. She further came a week later for a follow-up, during which residual sensory deficits remained. However, the patient then sought to do the follow-ups at a nearby center. 

## Discussion

Given its rarity and heterogeneity of clinical features, infarction of the artery of Percheron (AOP) poses a challenge both in terms of diagnosis and therapy. Occlusion of this unusual arterial trunk is frequently underdiagnosed in clinical practice, mostly due to its atypical and/or uncommon presentation and similarity with other neurological disorders [10-12]. This case illustrates the challenge in diagnosing AOP infarction in an adult with no previously identified vascular risk factors but a strong family history of cardiovascular disease.

Pathophysiology and anatomical considerations

**Figure 3:**
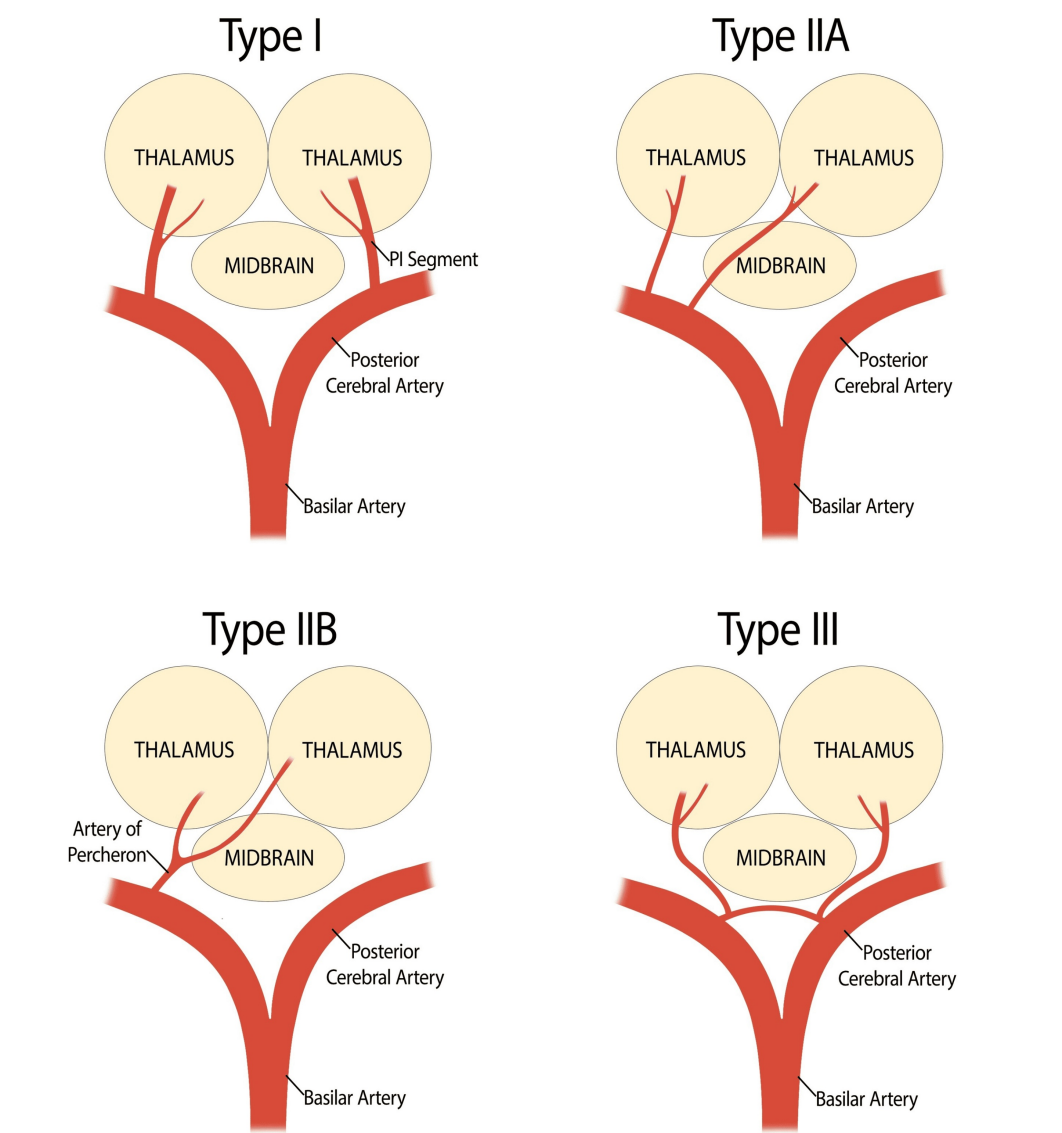
Paramedian thalamic vasculature showing the artery of Percheron, an anatomical variant (Type IIb) This figure is the authors' own creation.

The thalamus (especially the paramedian section) is important for consciousness, processing of the senses, and coordination of movements. The AOP is an infrequent anatomical variant from the P1 division of the posterior cerebral artery (PCA), which bifurcates to provide blood flow to both the paramedian thalami and, occasionally, the rostral midbrain (Figure 3) [1-3,13].

Bilateral thalamic infarctions are rare entities because unilateral lesions (both small and large) predominate with thalamic strokes due to the dual arterial supply from different PCAs. The AOP is more susceptible to the development of ischemia, as it supplies blood to both thalami via a single arterial trunk. This anatomical disposition renders the superimposed lesions typically bilateral and symmetric when visualized by imaging methods [3,13].

Clinical presentation

The clinical presentation of AOP infarction is heterogeneous, ranging from mild cognitive and behavioral alterations to severe neurological deficits such as coma, akinetic mutism, and vertical gaze palsy [5,14]. The classic triad of altered mental status, vertical gaze palsy, and memory impairment is often pathognomonic, but not all features may be present in every case [14,15]. The present case illustrates that AOP infarction may manifest in more subtle yet insidious ways, especially in the earlier stages of the disease, as the patient presented with transient visual disturbances, generalized fatigue, and decreased responsiveness. Nevertheless, bilateral thalamic infarction can resemble other neurological conditions, such as basilar artery thrombosis, Wernicke's encephalopathy, autoimmune encephalitis, and central pontine myelinolysis [7,10]. This diagnostic uncertainty may delay the provision of adequate treatment, thus negatively impacting patient outcomes [7].

Diagnostic challenges

The cornerstone of diagnosis for AOP infarction is magnetic resonance imaging (MRI) with diffusion-weighted imaging (DWI), the latter characteristically demonstrating symmetrical hyperintensities in the bilateral paramedian thalami [5]. Symmetrical restriction of diffusion was observed in the bilateral medial thalami of our patient on MRI, although the magnetic resonance angiography (MRA) was unremarkable.

However, in some circumstances, initial imaging can be inconclusive and so may require repeat imaging and the use of complementary modalities, including magnetic resonance angiography (MRA) or computed tomography angiography (CTA), to visualize the occlusion. Guillaume Cassourret et al. described a 64-year-old Caucasian man who was comatose, and the initial MRI at 95 minutes was unremarkable. A CT scan taken 48 hours later was consistent with an artery of Percheron infarct. The patient was given intravenous heparin and other supportive measures and made a good recovery [16]. These highlight the necessity of correlating diagnostic imaging with clinical findings [2,17-19].

Imaging technology has improved but can still lead to misdiagnosis or a delayed diagnosis, especially in younger stroke patients without traditional vascular risk factors [4,5,14].

Hypertension, diabetes mellitus, and hyperlipidemia are classical risk factors for AOP infarction as they contribute to atherosclerosis and embolic phenomena. In contrast, the etiology in the younger stroke population may be associated with rarer mechanisms, including genetic predisposition, cardioembolic sources such as patent foramen ovale, and systemic autoimmune disease [12,18]. Hence, a detailed clinical assessment, including family history and necessary investigations, is crucial to detect risk factors that might affect management.

Management and prognosis

The main goals of AOP infarction management should be to target cerebral perfusion restoration and prevent secondary sequelae. For patients who present within the therapeutic window, intravenous thrombolysis with recombinant tissue plasminogen activator (rtPA) is considered the standard of care. However, owing to the rarity of AOP infarcts, little is known regarding guidelines for thrombolysis in this population, and decisions are often guided by general stroke management algorithms [5,14,20].

In cases with delayed presentation precluding thrombolytic therapy, a conservative approach is mandated with antiplatelet agents, anticoagulation, and neuroprotective means [20,21]. Though our patient achieved good recovery with residual sensory deficits at the first follow-up visit, regular follow-ups are necessary for further evaluation of the existing deficits and occurrence of any new symptoms. This highlights the importance of early supportive care and rehabilitation to improve outcomes.

The prognosis for AOP infarction is quite heterogeneous and is based on the degree of neurological involvement, prompt diagnosis, and other comorbidities. Some patients recover with few residual deficits, but others have persistent cognitive and functional impairments that impact their quality of life [4,6,15,18]. Donohoe C et al. reported the case of a 56-year-old African American female who was unresponsive at presentation with a GCS score of 7. Due to the nonspecific presentation and severity of the stroke, the patient, unfortunately, had a poor prognosis and later did not achieve any significant improvement clinically [4].

Family history and genetic predisposition

In young stroke patients without previously identified classical vascular risk factors, investigation of alternative etiologies, including genetic susceptibility, cardioembolic sources, and systemic autoimmune diseases, should be thought of [9]. The history of stroke and myocardial infarction in our patients' immediate family may have strengthened the hereditary cause of AOP infarction. In such cases, genetic screening and cardiovascular risk assessment may be indicated to expose underlying predispositions and undertake preventive measures [12,17]. Nevertheless, financial constraints can have limitations on investigations and are a barrier to finding the exact cause. 

In many cases, the clinical course is further complicated by the misdiagnosis of patients as having metabolic or psychiatric disorders. Thus, a high level of suspicion is needed among clinicians, when patients present with symptoms suggestive of bilateral thalamic syndromes, especially with a history of cardiovascular risk factors [21].

## Conclusions

This case highlights the importance of being aware of potential differential diagnoses as well as the necessity for increased clinical suspicion and a structured diagnostic workup when assessing bilateral thalamic lesions. Differential diagnosis is challenging, but advanced neuroimaging techniques, such as diffusion-weighted imaging (DWI) and magnetic resonance angiography (MRA), aid in accurate diagnosis and management in such complex cases.

The practicality of intravenous thrombolysis, the mainstay treatment for acute ischemic stroke, is often limited in AOP infarction because of late patient presentation and diagnostic uncertainty. Thus, management with supportive care, neuroprotective approaches, and rehabilitation are important for maximizing recovery and minimizing residual deficits. Limited research has been conducted on this rare occurrence. More case reporting on its clinical spectrum can help in developing a better understanding of this condition, which can lead to improved diagnosis and timely treatment.

## References

[1] Percheron G (1973). The anatomy of the arterial supply of the human thalamus and its use for the interpretation of the thalamic vascular pathology. Z Neurol.

[2] Amin OS, Shwani SS, Zangana HM, Hussein EM, Ameen NA (2011). Bilateral infarction of paramedian thalami: a report of two cases of artery of Percheron occlusion and review of the literature. BMJ Case Rep.

[3] Lazzaro NA, Wright B, Castillo M (2010). Artery of percheron infarction: imaging patterns and clinical spectrum. AJNR Am J Neuroradiol.

[4] Donohoe C, Nia NK, Carey P, Vemulapalli V (2022). Artery of Percheron infarction: a case report of bilateral thalamic stroke presenting with acute encephalopathy. Case Rep Neurol Med.

[5] Morais J, Oliveira AA, Burmester I, Pires O (2021). Artery of Percheron infarct: a diagnostic challenge. BMJ Case Rep.

[6] Khanni JL, Casale JA, Koek AY, Espinosa Del Pozo PH, Espinosa PS (2018). Artery of Percheron infarct: an acute diagnostic challenge with a spectrum of clinical presentations. Cureus.

[7] Aaron S, Mani S, Prabhakar AT, Karthik K, Patil AK, Babu PS, Alexander M (2015). Stuck with a drowsy patient, evoke the Percheron. Neurol India.

[8] Brown DL, Levine DA, Albright K (2021). Benefits and risks of dual versus single antiplatelet therapy for secondary stroke prevention: a systematic review for the 2021 guideline for the prevention of stroke in patients with stroke and transient ischemic attack. Stroke.

[9] Stack CA., John WC (2021). The Clinical Approach to Stroke in Young Adults. Stroke [Internet].

[10] Renard D, Castelnovo G, Campello C (2014). Thalamic lesions: a radiological review. Behav Neurol.

[11] Ahmed RA, Dmytriw AA, Regenhardt RW, Leslie-Mazwi TM, Hirsch JA (2023). Posterior circulation cerebral infarction: a review of clinical, imaging features, management, and outcomes. Eur J Radiol Open.

[12] American Journal of Neuroradiology (2009). Uncommon Causes of Stroke. Uncommon Causes of Stroke.

[13] Lahnine G, Abdourabbih Y, El Bouardi N (2024). Bilateral thalamic infarcts: Percheron territory. Radiol Case Rep.

[14] Musa J, Rahman M, Guy A (2021). Artery of Percheron infarction: a case report and literature review. Radiol Case Rep.

[15] Snyder HE, Ali S, Sue J, Unsal A, Fong C, Deng Z (2020). Artery of Percheron infarction with persistent amnesia: a case report of bilateral paramedian thalamic syndrome. BMC Neurol.

[16] Cassourret G, Prunet B, Sbardella F, Bordes J, Maurin O, Boret H (2010). Ischemic stroke of the artery of Percheron with normal initial MRI: a case report. Case Rep Med.

[17] Li J, Ge J, Yang S, Yao G (2023). Clinical review and analysis of artery of Percheron infarction. IBRO Neurosci Rep.

[18] Vinod KV, Kaaviya R, Arpita B (2016). Artery of Percheron infarction. Ann Neurosci.

[19] Kichloo A, Jamal SM, Zain EA, Wani F, Vipparala N (2019). Artery of Percheron infarction: a short review. J Investig Med High Impact Case Rep.

[20] Li X, Agarwal N, Hansberry DR, Prestigiacomo CJ, Gandhi CD (2015). Contemporary therapeutic strategies for occlusion of the artery of Percheron: a review of the literature. J Neurointerv Surg.

[21] Gurley KL, Edlow JA (2019). Avoiding misdiagnosis in patients with posterior circulation ischemia: a narrative review. Acad Emerg Med.

